# Bible Overclaiming and Intimate Partner Violence

**DOI:** 10.1177/08862605231225518

**Published:** 2024-01-10

**Authors:** Jacqueline Lechuga, Daniel N. Jones

**Affiliations:** 1 The University of Texas at El Paso, USA; 2 University of Nevada, Reno, USA

**Keywords:** overclaiming, religion, intimate partner violence, religiosity, violence

## Abstract

Religion has had a mixed impact on society, with some followers engaging in violent behavior. It remains unclear why some followers perpetrate violence and others are peaceful. We argue that religious overclaiming is one facet of religion to be considered when trying to understand the relationship between religion and violence. Across two studies (*N* = 551), we tested the hypothesis that a higher tendency to overclaim knowledge of the Christian Bible would be associated with higher perpetration of intimate partner violence (IPV). We also tested the hypotheses that men who overclaim would be most likely to engage in the perpetration of IPV, and that higher religiosity would attenuate the effects of religious overclaiming. In both studies, participants completed a measure of religious overclaiming, reported on their perpetration of IPV, and reported their religiosity. Our findings across both studies indicated that Bible overclaiming was associated with greater perpetration of IPV. Further, Study 1 found that those high in Bible overclaiming (especially men) engaged in the most perpetration of IPV. However, this gender-based finding did not replicate in Study 2. Both studies found that religiosity was unassociated with the perpetration of IPV. Our results provide evidence that Bible overclaiming is related to the perpetration of IPV. Specifically, individuals who claim to know religious concepts that do not exist are associated with a higher risk for IPV.

Religious teachings have messages of both peace and violence. For example, the Christian Bible preaches love for one another and equity in treatment (e.g., van Eck & Kloppenborg, 2015). However, among other examples of gender inequality, the Bible also emphasizes that women should submit to their husbands (e.g., Perry & McElroy, 2020). Interpretations of religious texts vary based on personal biases (Perry & McElroy, 2020). Thus, some may interpret this statement as a responsibility imparted upon men to care for women, whereas others may interpret this statement as a license for men to treat women as property.

However, research is needed to distinguish between these two types of religious followers. Although both types of followers may be highly religious, overconfidence in religious knowledge may lead some religious followers to interpret scriptures in ways that are self-serving (Gebauer et al., 2017; Jones et al., 2020). Self-serving interpretations allow religious teachings to become distorted with prejudices. Research on Christian overclaiming has argued that those who religiously overclaim are also egotistical about their contributions to a community, their kindness, and their charity (Gebauer et al., 2017). Similarly, those who overclaim religious knowledge are more likely to support violence in the name of God (Jones et al., 2020). We expect similar findings when testing the relationship between religious overclaiming and violence that occurs in the context of a romantic relationship. Religious teachings that subjugate women would likely give rise to entitlement among male overclaimers, and a sense of obligation in male non-overclaimers. This entitlement would then be associated with increased perpetration of violence in a romantic relationship among overclaimers (vs. non-overclaimers).

The purpose of this investigation is to determine if there is a link between Bible overclaiming and intimate partner violence (IPV). Previous research has found that religious overclaiming is linked with supporting terrorism-like violence in the name of God across different religions (Jones et al., 2020). Building on this research, we test how religious overclaiming influences the perpetration of violence in an interpersonal context. Our paper is structured in the following way. First, we discuss the paradox that research findings on the link between religion and support/perpetration of violence present. Second, we discuss religion as a multifaceted construct and underscore religious overclaiming as a facet of religion to consider when seeking to understand this paradox. Third, we discuss conflicting research findings on the role of religion and violence in romantic relationships. Finally, we present and discuss the findings of two studies that demonstrate a link between religious overclaiming and IPV. Further, we briefly discuss the controversies surrounding overclaiming behavior. We conclude this section with limitations, implications, and future directions.

## Literature Review

All religions offer their devotees a system of beliefs that influence their thoughts and behaviors in day-to-day life. Religion motivates positive actions as well as negative actions in people. Consider, for instance, the Missionaries of Charity and the Army of God. The Missionaries of Charity are dedicated to serving the poorest of the poor irrespective of social class, creed, color, or religion (Missionaries of Charity, n.d.). The Army of God is an anti-abortion movement that openly supports violent acts to stop abortion (Army of God, n.d.). Both are based on the Christian faith, and their missions are motivated by verses that exist in the same Bible. Nonetheless, their actions are opposite in terms of societal effect. The Missionaries of Charity encourage inclusivity whereas the Army of God encourages intolerance. The discrepancy in the type of actions stemming from religion, as underscored by the example above, has been a reason for debate among researchers. No religion is inherently violent. Nonetheless, there are numerous investigations that place religion at the forefront of the perpetration of violence or other negative actions (Beller & Kröger, 2018; Blogowska & Saroglou, 2011; Bushman et al., 2007; Henne, 2012). Religion is a multifaceted construct, and the study of its different facets is likely to be driving the inconsistencies in research findings. Some facets of religion are associated with increased violence, whereas other facets of religion are not (Allport & Ross, 1967; Beller & Kröger, 2018; Beller, Kröger, & Hosser, 2021; Preston & Ritter, 2013). In this investigation, we direct attention to a facet of religion—religious overclaiming—that has been understudied when investigating the relationship between religion and violence.

## The Positive and Negative Influence of Religion

Religion offers numerous benefits for the self and interpersonal interactions. People turn to their religion to find the power to deal with life problems such as lack of health (Johnston et al., 2021), the loss of a loved one (Wortmann & Park, 2008), or overall stress (Whitehead & Bergeman, 2020). Additionally, people use religion for self-improvement such as recovering from an addiction (Lovett & Weisz, 2021) or cultivating positive personality traits (Lambert & Dollahite, 2006; Lovett & Weisz, 2021). Further, religion encourages positive relationships with others such as spending more quality time with family and engagement in altruistic behavior (Pew Research Center, 2016). However, people also turn to their religion to justify others or their negative actions. For example, religion is associated with having prejudices toward others who do not follow their religious norms (Blogowska & Saroglou, 2011) as well as support for terrorist strategies (e.g., suicide) (Beller & Kröger, 2018; Henne, 2012). One’s perpetration of violence is also rationalized with religion. For instance, believers and non-believers behaved more aggressively after being exposed to a violent passage that they believed came from the Bible rather than another source (e.g., an ancient scroll) and when they believed the violence had been sanctioned by God (Bushman et al., 2007). The polarity in the findings of this literature gives rise to a paradox. How can religion have opposing motivations for good and bad actions?

## Religion as a Multifaceted Construct

The discrepancy in findings regarding the relationship between religion and violence is most likely due to the multifaceted nature of religion. A thorough explanation of all facets of religion is beyond the scope of this paper. However, a few examples are included to highlight how different facets lead to variations in research findings and point to the importance of understanding other facets of religion.

### Religiosity Versus Spirituality

Distinguishing between religiosity and spirituality is important as they can lead to different findings. An important distinction to make is that religiosity is a group practice whereas spirituality is an individual practice (Leach et al., 2008). Being religious is linked to identifying with a group (i.e., religious affiliation). Therefore, religiosity (vs. spirituality) is more restrictive, and it is more bound to be influenced by ingroup and outgroup biases. For instance, when people are primed with religion (vs. God) people are more likely to be prosocial with ingroup members than outgroup members (Preston & Ritter, 2013). Similarly, perceiving their group (i.e., religion) to be threatened increases support for extremist violence (Beller & Kröger, 2018).

### Religious Motivations

Religious engagement can be divided into actions that are intrinsically motivated and those that are extrinsically motivated. People with intrinsic motivation have an innate interest in their religion and practices whereas people with extrinsic motivations have external reasons to be interested (Allport & Ross, 1967). Individual practices such as private prayer are thought to be intrinsically motivated whereas social practices like attendance at religious sermons are thought to be extrinsically motivated. Past work comparing different religious attitudes has suggested that people who are driven by intrinsic motivation tend to display relatively lower levels of prejudice than other types of motivation (Allport & Ross, 1967). Another set of studies using a representative multinational data set (i.e., The World’s Muslims) showed that engagement in extrinsically motivated practices (i.e., mosque attendance) was associated with increased support for extremist violence (i.e., suicide bombing; Beller & Kröger, 2018) and honor killings (i.e., the murder of a man/woman for engaging in premarital sex or adultery; Beller et al., 2021). Nonetheless, it is important to note that these findings may also be influenced by group dynamics rather than exclusively reflecting a person’s religious beliefs or attitudes (Beller et al., 2021). Additionally, intrinsic religious motives can be divided into further facets that can be used to predict aggression. For example, people who internalize their religiosity because of sincere enjoyment (i.e., identified religious self-regulation) were less likely to perpetrate aggression compared to people who internalize religion because of rewards or punishments (i.e., introjected religious self-regulation; Renzetti et al., 2017). Finally, people with quest motivation—driven by openness and exploration—are less likely to endorse negative beliefs such as rape myth acceptance (Ensz & Jankowski, 2020). In contrast, people with religious fundamentalism—driven by rigorous adherence to rules—show the opposite trend (Ensz & Jankowski, 2020).

The findings discussed above show that perhaps the question is not whether religion causes violence, but what facets of religion encourage people to use religion as a justification for violence. For decades, the religion-violence paradox has baffled scholars inside and outside the field of psychology. It appears that for every positive effect attributed to religion, there is a corresponding trade-off that must be acknowledged. Although our understanding of this paradox has increased, the numerous facets of religion that have been and continue to be revealed hint at the work that remains to be undertaken. We propose that religious overclaiming is another facet of religion to be considered when investigating the relationship between religion and violence.

## Overclaiming of Religious Knowledge

Overclaiming is the tendency to assert familiarity with concepts that could not possibly be known (Paulhus & Bruce, 1990). The act of overclaiming is a *behavior* and can be objectively measured (see Methods section). Overclaiming is more likely to occur for domains that hold significance for one’s self-concept, such as religion (Gebauer et al., 2017). Although people are increasingly identifying as religiously unaffiliated, most people in the United States (approximately 75%) still identify with a religion (Public Religion Research Institute, 2021). Therefore, people may engage in religious overclaiming—asserting familiarity with non-existent Biblical characters or events. Limited research has been conducted on religious overclaiming. We know that—unlike secular overclaiming—religious overclaiming is positively associated with religious violence (Jones et al., 2020). Thus, there is evidence that claiming to know more about one’s religion than one does is a facet of religion associated with religiously motivated violence.

Despite being a robust predictor for a variety of outcomes, overclaiming has been difficult to theoretically define (Bensch et al., 2019). There are multiple theoretical perspectives that attempt to explain the phenomenon of overclaiming (Goecke et al., 2020). Not one perspective, however, fully explains the phenomenon of overclaiming (Goecke et al., 2020), primarily because overclaiming does not neatly fall in any one particular dimensional space (Bensch et al., 2019). In this investigation, we are only interested in the overall behavior of overclaiming and not in its underlying mechanism (i.e., why someone engages in overclaiming behavior). Our discussion, however, engages more with this topic. Overall, we argue that exaggerating one’s expertise in religious knowledge can be destructive and result in negative interpersonal relationships. More so if that exaggerated, inaccurate knowledge is being used to make decisions or behave in a specific way (i.e., perpetrating violence).

## Religiously Motivated Violence in Romantic Relationships

IPV includes physical, sexual, or psychological violence/aggression committed by a current or former intimate partner (Breiding et al., 2015). Unfortunately, IPV is a major societal problem, with one in four women and one in nine men reporting being victims of IPV (National Coalition Against Domestic Violence [NCADV], 2020). In the context of romantic relationships, the link between religion and violence is also inconsistent. Some studies support the idea that religion benefits a romantic relationship. It can help couples prevent problems in their relationship, resolve conflicts, and work on reconciliation (Lambert & Dollahite, 2006). It can also protect against IPV. Greater attendance at religious services is associated with a lower likelihood of perpetrating violence toward a romantic partner (Ellison & Anderson, 2001; Kim, 2018). If IPV is a problem in the relationship, Muslims and Christians use their religion to find the strength to deal with it (Ghafournia, 2017; Simonič, 2021) or to begin the process of leaving the relationship (Simonič, 2021). Other studies, however, support the idea that religion can harm a romantic relationship. Victims of IPV often report their partners using religion to excuse their behaviors, request behaviors from them, and/or keep them in the relationship (Cares & Cusick, 2012; Ghafournia, 2017). Further, victims of IPV report that their religious community and leaders may deny the occurrence of IPV and encourage them to stay in those relationships (Cares & Cusick, 2012; Ghafournia, 2017). The discrepancy in these findings parallels those previously discussed for the relationship between religion and violence. Thus, IPV is an ideal context to explore the link between religious overclaiming and religiously motivated violence.

## Current Study

This investigation extends our understanding of the association between religion and violence by exploring the correlation between religious overclaiming and IPV. We tested this idea in the context of romantic relationships because violence in romantic relationships is common. Our primary interest was to test the hypothesis that greater religious overclaiming would be associated with greater perpetration of IPV. Our secondary interest was to test gender and religiosity as moderators. We tested whether men with higher (vs. lower) religious overclaiming report greater perpetration of violence toward a romantic partner. We also tested if the association between religious overclaiming and violence in a romantic relationship would be lower among people with greater religiosity. All our hypotheses were pre-registered, and our data and the syntax used to analyze the data can be found in our Open Science Framework (OSF) profile (https://osf.io/cfxdm/?view_only=3a113312e249487ab196a09e187d3eb0).

## Study 1—Methods

### Participants

A total of 293 participants were recruited for this study, but 15 participants were excluded from data analyses because they did not identify with male or female as their gender.^1^ Our final sample consisted of 278 participants (73.4% Female, 3.6% Black/African Heritage, 5.4% White/European Heritage, 84.4% Hispanic, 2.2% South Asian, 0.7% Middle Eastern/Arabic, 0.4% Native North American, 3.3% Other). Participants ranged in age from 18 to 35 (*M* = 19.89, *SD* = 2.64), were college students, and were given class credit for their participation.

### Materials and Procedure

Participants completed a battery of measures that included assessments for other studies unrelated to this one. Measures relevant to this study are described below. All measures were available in English and Spanish. All means, standard deviations, and reliability scores for all measures in Study 1 and Study 2 are reported in Table 1.

**Table 1.** Correlations Among Variables for Study 1 and Study 2.

Measured Variables		1	2	3	4
1. Accuracy (*d′*)^a^		—	−.07	−.16*	**−.003**
2. Religiosity		−.05	—	.03	**−.07**
3. Perpetration		−.22*	.03	—	−.14*
4. Gender		−.12*	−.19*	.06	—
Study 1	*M*	.93	3.64	.15	—
	*SD*	.65	1.82	.26	—
	α	—	—	.89	—
Study 2	*M*	.96	3.41	.60	—
	*SD*	.65	1.76	.44	—
	α	—	—	.97	—

*Note.* Results reported below the diagonal belong to Study 1. Results reported above the diagonal belong to Study 2.

The total number of observations ranged from 274 to 278 in Study 1. The total number of observations ranged from 263 to 273 in Study 2. Correlations in bold represent associations that were not replicated from Study 1.

a Higher scores of *d′* indicate lower overclaiming whereas lower scores of *d′* indicate higher overclaiming.

* *p* < .05.

### Bible Overclaiming Questionnaire

The Bible Overclaiming Questionnaire (B-OCQ) is a 36-item questionnaire that assesses overclaiming of Christian religious knowledge (Jones et al., 2020). Participants rated their familiarity with characters/events from the Bible using a seven-point Likert scale (0 = *Never heard of it* to 6 = *Extremely familiar*). Some of the characters/events are real (e.g., “Cain and Abel”), but others are fake (e.g., “The Journey of Aruk”). A score of overclaiming is computed using signal detection theory (SDT; Stanislaw & Todorov, 1999). In brief, SDT separates responses of true and false items into four categories: True recognitions (knowing a true item), True rejections (not knowing a fake item), False rejections (not knowing a true item), and False recognitions (claiming to know a fake item). In this investigation, recognition corresponds to a participant reporting familiarity with an item and rejection corresponds to a participant reporting no familiarity with an item. SDT provides several metrics relevant to overclaiming; here, we focus on the most used metric, *d′* (Paulhus et al., 2003). The metric of *d′* is best described as one’s ability to discriminate between true and false items. Therefore, the metric of *d′* is not merely a simple measure of accuracy (i.e., the number of correct items). Rather, *d′* is calculated by taking into consideration the participants’ performance in true recognitions (knowing a true item) *and* false recognitions (claiming to know a fake item). Thus, people scoring lower on *d′* fail to minimize errors. Consequently, higher scores of *d′* indicate lower overclaiming whereas lower scores of *d′* indicate higher overclaiming.

### Conflict Tactics Scale Revised Short Form

The Conflict Tactics Scale (CTS) Revised Short Form is a 20-item scale that assesses the usage of five conflict tactics: conflict negotiation, physical assault, injury, sexual coercion, and psychological aggression (Straus & Douglas, 2004). Half of the items inquire about the frequency of behaviors the participant engaged in (e.g., “I insulted or swore or shouted or yelled at my partner”) and the other half of the items inquire about the frequency of behaviors the participant’s romantic partner engaged in (e.g., “My partner insulted or swore or shouted or yelled at me”). Participants reported the number of times these behaviors occurred within the last 12 months using a six-point Likert scale (1 = *One in the past year* to 6 = *More than 20 times in the past year*). Participants were also given the option to indicate that these behaviors had never happened before or that these behaviors had happened before but not within the time frame they were asked to recall. If participants were not in a romantic relationship at the time of the study, they were asked to think of their last romantic partner. Items related to conflict negotiation and items assessing victimization were not used for analyses. There has been debate over the scoring of the CTS scale. For example, Shorey et al. (2012) argued that among the three most popular scoring methods (*sum*: adding the items together; *frequency*: scores of how often particular acts occurred by using the midpoint for each response, or *variety*: total number of items that were positively endorsed at all), *variety* scoring made the most sense with respect to distributions and limitations of memory recall. Thus, we used variety scoring to create a perpetration score.

### Religiosity

One item was included in the demographic form to assess participants’ level of religiosity. Participants were asked “How religious are you?” (0 = *Not at all* to 6 = *Extremely*). Religiosity was analyzed as a continuous variable because none of our participants selected zero as their response. This was also true for Study 2.

## Study 1—Results

### Data Analytic Strategy

Initial analyses consisted of correlational tests to assess the association among variables. We next examined the role of overclaiming and gender in the perpetration of IPV in a hierarchical linear regression. In Step 1, perpetration of IPV was the outcome variable and overclaiming and gender (contrast coded 1 = men, −1 = women) were the predictors. In Step 2, the interaction between overclaiming and gender was entered as an additional predictor. This analytical strategy was used to analyze data collected for both Study 1 and Study 2.

### Correlations

Correlations among the main study variables can be seen in Table 1. There was no association between accuracy in Bible knowledge and religiosity (*r* = −.05, *p* = .456). However, lower accuracy in Bible knowledge (i.e., higher overclaiming) was associated with higher levels of IPV perpetration (*r* = −.22, *p* < .001). Overall, men had lower accuracy (i.e., higher overclaiming) (*r* = −.12, *p* = .041), and lower levels of religiosity (*r* = −.19, *p* = .001). Finally, gender (*r* = .06, *p* = .326) and being religious (*r* = .03, *p* = .684) did not correlate with IPV perpetration.

### Overclaiming and Gender on Perpetration of IPV

We set up a hierarchical linear regression to determine if gender moderated the overclaiming–IPV link. In Step 1 (*F* [2, 271] = 7.15, *p* < .001, *R*^2^ = .05), higher accuracy in Bible knowledge (i.e., lower overclaiming) resulted in lower perpetration of IPV (*B* = −0.09, 95% CI [−0.13, −0.04], β = −.22, *p* < .001), whereas gender was unassociated with perpetration of IPV (*B* = 0.01, [−0.02, 0.05], β = .04, *p* = .538). In Step 2 (*F* [3, 270] = 7.17, *p* < .001, *R*^2^ = .07), we found that accuracy in Bible knowledge still had a negative main effect on perpetration of IPV (*B* = −0.12, [−0.18, −0.07], β = −.31, *p* < .001), gender remained unassociated with perpetration of IPV (*B* = 0.004, [−0.03, 0.04], β = .02, *p* < .807), but there was a significant interaction between accuracy in Bible knowledge and gender (*B* = −0.05, [−0.08, −0.01], β = −0.18, *p* = .009). Simple slopes analyses revealed that men with lower accuracy (i.e., a higher overclaiming) were more likely to engage in the perpetration of IPV (*B* = −0.13, [−0.19, −0.07], β = −0.49; *p* < .001). However, there was no effect of gender on perpetration of IPV at high levels of accuracy (i.e., lower overclaiming; *B* = −0.03, [−0.07, 0.001], β = −.13, *p* = .057).

### Overclaiming and Religiosity on Perpetration of IPV

We also set up a hierarchical linear regression to determine if religiosity moderated the overclaiming–IPV link. In Step 1 (*F* [2, 271] = 6.98, *p* = .001, *R*^2^ = .05), higher accuracy in Bible knowledge (i.e., lower overclaiming) resulted in lower perpetration of IPV (*B* = −0.09, 95% CI [−0.14, −0.04], β = −.22, *p* < .001), whereas religiosity was unassociated with perpetration of IPV (*B* = 0.002, [−0.02, 0.02], β = .01, *p* = .812). In Step 2 (*F* [3, 270] = 5.79, *p* < .001, *R*^2^ = .06), the effects for accuracy of Bible knowledge (*B* = −0.09, [−0.13, −0.04], β = −.22, *p* < .001) and religiosity (*B* = 0.001, [−0.02, 0.02], β = .004, *p* = .945) on perpetration of IPV remained the same. The interaction between accuracy in Bible knowledge and religiosity was not significant (*B* = 0.03, [−0.002, 0.06], β = .11, *p* = .070).

## Study 2—Methods

### Participants

A total of 297 participants were recruited for this study, but 24 participants were excluded from data analyses because they did not identify with male or female as their gender. Our final sample consisted of 273 participants (73.1% Female, 5.2% Black/African Heritage, 44.6% White/European Heritage, 36.9% Hispanic, 5.2% East Asian, 1.5% South Asian, 0.4% Middle Eastern/Arabic, 0.7% Native North American, 5.5% Other). Participants ranged in age from 18 to 50 (*M* = 21.46, *SD* = 3.98) were college students and were given class credit for their participation. People who participated in Study 1 were not eligible to participate in this study.

### Materials and Procedure

The materials and procedure of this study were almost identical to those in Study 1. Participants completed a battery of measures that included assessments for other studies unrelated to this one. For this study, participants completed the B-OCQ, CTS, and responded to the religiosity item. All participants in Study 2, however, completed our measures in English.^2^

## Study 2—Results

### Correlations

Correlations among the main study variables can be seen in Table 1. There was no association between accuracy in Bible knowledge and religiosity (*r* = −.07, *p* = .256). However, lower accuracy in Bible knowledge (i.e., higher overclaiming) was associated with higher levels of IPV perpetration (*r* = −.16, *p* = .013). Finally, religiosity was not associated perpetration of IPV (*r* = .03, *p* = .603). These results replicate the findings of Study 1. Unlike Study 1, however, there was no association between accuracy in Bible knowledge and gender (*r* = −.003, *p* = .956), no association between religiosity and gender (*r* =* −*.07, *p* = .270), and there was a significant association between gender and the perpetration of IPV such that women reported higher perpetration of IPV (*r* =* −*.14, *p* = .026).

### Overclaiming and Gender on Perpetration of IPV

We conducted the same hierarchical linear regression as in Study 1 to determine if gender moderated the overclaiming–IPV link. In Step 1 (*F* [2, 255] = 6.5, *p* = .002, *R*^2^ = .05), higher accuracy in Bible knowledge (i.e., lower overclaiming) resulted in lower perpetration of IPV (*B* = −0.11, 95% CI [−0.19, −0.03], β = −.16, *p* = .011), and gender had a significant main effect on perpetration of IPV (*B* = −0.08, [−0.14, −0.02], β = −.16, *p* = .011) such that women reported higher perpetration. In Step 2 (*F* [3, 254] = 4.34, *p* = .005, *R*^2^ = .05), we found that accuracy in Bible knowledge still had a negative main effect on perpetration of IPV (*B* = −0.11, [−0.20, −0.02], β = −.16, *p* = .013), and women reported higher perpetration (*B* = −0.08, [−0.14, −0.02], β = −.16, *p* = .011). However, unlike Study 1, there was no interaction between accuracy and gender in predicting IPV (*B* = −0.01, [−0.06, 0.05], β = −.02, *p* = .806).

### Overclaiming and Religiosity on Perpetration of IPV

We also set up a hierarchical linear regression to determine if religiosity moderated the overclaiming–IPV link. In Step 1 (*F* [2, 255] = 3.15, *p* = .045, *R*^2^ = .02), higher accuracy in Bible knowledge (i.e., lower overclaiming) resulted in lower perpetration of IPV (*B* = −0.11, 95% CI [−0.19, −0.02], β* = −*.15, *p* = .014) whereas religiosity was unassociated with perpetration of IPV (*B* = 0.003, [−0.03, 0.03], β = .01, *p* = .858). In Step 2 (*F* [3, 254] = 2.87, *p* = .037, *R*^2^ = .03), the effects for accuracy of Bible knowledge (*B* = −0.11, [−0.19, −0.02], β = −.16, *p* = .012) and religiosity (*B* = 0.00, [−0.03, 0.03], β = −.001, *p* = .988) on perpetration of IPV remained the same. The interaction between accuracy in Bible knowledge and religiosity was not significant (*B* = 0.04, [−0.01, 0.09], β = .09, *p* = .132).

## General Discussion

The role of religion in the perpetration of violence can better be understood by identifying which facets of religion are associated with violence. We were primarily interested in testing how overclaiming religious knowledge was associated with the perpetration of IPV. Participants completed a measure of religious overclaiming, reported on their perpetration of IPV, and reported their religiosity. Across both studies, we found that higher religious overclaiming was associated with greater perpetration of IPV. We also tested if and how gender and religiosity would change this association. In this investigation, we are solely interested in overclaiming behavior and how it is associated with IPV. A test for the underlying mechanism of overclaiming behavior was not conducted. Nonetheless, we have included a brief section discussing the importance of investigating these mechanisms. Although our findings associating religious overclaiming with IPV are strong, there were mixed findings for gender as a moderator.

### Greater Perpetration of IPV as a Result of Religious Overclaiming

People with a higher tendency to overclaim reported greater perpetration of IPV toward their romantic partners as predicted. This is consistent with research findings that have demonstrated that greater religious overclaiming results in greater support for religious violence (Jones et al., 2020). Similarly, other forms of overclaiming have been associated with undesirable outcomes. For instance, higher overclaiming in an academic setting is associated with lower course grades (Paulhus & Dubois, 2014), and higher overclaiming in a professional setting is associated with engaging in deviant behavior (Dunlop et al., 2020). Hence, the tendency to overclaim appears to be maladaptive and can lead to poor outcomes for the self and others in a diversity of domains. In this investigation, we find strong evidence that exaggerated and/or inaccurate knowledge about one’s religion is linked to increased aggression displayed within a romantic relationship. Although tested in the context of a romantic relationship, an important implication of our results is that they provide a potential explanation as to why there are mixed findings in the literature for the religion-violence relationship. Specifically, our findings demonstrate that religious overclaiming is an important facet of any religion that must be considered when predicting religiously motivated violence.

### Men Who Overclaim Show More Violence Toward Their Romantic Partners

In Study 1, as predicted, men who overclaimed reported greater perpetration of IPV toward a romantic partner. Different national and international statistics place women (vs. men) as most likely to be a victim of IPV and other related forms of violence, whereas they place men (vs. women) as most likely to be a perpetrator (NCADV, 2020; Violence Policy Center [VPC], 2021; United Nations Office on Drugs and Crime [UNODC], 2019). Further, religion appears to have a greater influence on men’s perpetration of violence. Bushman et al. (2017) found that men were more likely to be aggressive when they believed the violence had been sanctioned by God. Beller and Kröger (2018) also found that men were more likely to support others’ engagement in extremist violence. Thus, it appears that men are more likely to use religion as a justification for their actions. In Study 2, this finding did not replicate, and instead, we found that women report higher perpetration of IPV. Although this finding goes against the national and international statistics on IPV (NCADV, 2020; VPC, 2021; UNODC, 2019), individual studies have shown that women at times do report more perpetration of IPV than men, and/or men report more victimization of IPV than women (Babcock et al., 2019; Simmons et al., 2015). It has also been documented that mutual violence is the most common form of IPV in romantic relationships (Anderson, 2002; Johnson et al., 2014). Overall, our findings for gender are mixed.

### Religiosity is Unassociated with Perpetration of IPV

It was predicted that high religiosity would attenuate the effects of religious overclaiming on the perpetration of IPV. However, our findings indicated that religiosity had no influence on violence perpetrated in a romantic relationship. This is inconsistent with some research studies. Greater engagement in one’s religion (e.g., attendance to religious services) lowers the perpetration of violence in a romantic relationship (Ellison & Anderson, 2001; Kim, 2018). In experimental studies, however, this has not been the case. Asking people to memorize Biblical passages or to meditate did not reduce the perpetration of violence (Leach et al., 2008). It is possible that the discrepancy in these findings is that in experimental studies these behaviors are prompted and thus do not reflect an intrinsic interest in religion. Having an intrinsic interest in being involved in religion may result in accurate learning of the religious teachings, whereas not having an intrinsic interest may lead to superficial knowledge that can be easily misinterpreted. In this investigation, we assessed religiosity with one item. This item does a poor job of distinguishing between intrinsic and extrinsic motivation for being religious. Arguably, intrinsic (vs. extrinsic) motivation should be associated with lower overclaiming behavior. Further, those with intrinsic motivation that reflects introjected religious self-regulation are different from those that reflect internal religious self-regulation (Renzetti et al., 2017). Thus, future research should examine the associations between religious overclaiming and different types of religious internalization. Similarly, quest and fundamentalist beliefs in religion predict different outcomes (Ensz & Jankowski, 2020). Thus, their associations with religious overclaiming should also be examined. More rigid people are seemingly more easily threatened when their religious beliefs are challenged, which may lead to religious aggression (Jones et al., 2020).

### Underlying Mechanisms of Overclaiming Behavior

In this investigation, we exclusively focused on the overall behavior of overclaiming and not on its underlying mechanism. Extensively reviewing the different perspectives for why someone engages in overclaiming behavior is out of the scope of this paper, but we want to give a nudge to the importance of investigating these nuances. The precise mechanism of overclaiming is still a topic of debate. The most prominent perspectives explain overclaiming behavior as (a) self-enhancement tendencies, (b) cognitive biases, (c) a proxy of cognitive abilities, or (d) a sign of creative engagement (Goecke et al., 2020). Nonetheless, a test of all these perspectives failed to provide strong evidence for a single explanation for why people engage in overclaiming behavior (Goecke et al., 2020). Closely related to understanding the underlying mechanism of overclaiming behavior is identifying which specific variables within each perspective are the strongest predictors of overclaiming behavior. Some of the variables that have been associated with overclaiming are narcissism (Gebauer et al., 2012; Goecke et al., 2020; Paulhus et al., 2003), intellectual humility (Zedelius et al., 2022), reconstruction biases (Müller & Moshagen, 2018), moral certainty (Vecina et al., 2015), right-wing authoritarianism (Jones et al., 2020) and social dominance orientation (Jones et al., 2020) to name a few. Finally, it is also important to consider the scoring method and the specific metric used to measure overclaiming. Different scoring methods and metrics could relate to different mechanisms. For instance, when using the SDT as a scoring method, the bias metric is related to self-enhancement traits (i.e., narcissism and self-esteem) but the accuracy metric is related to cognitive factors (i.e., crystalized intelligence; Paulhus et al., 2003). The perspective, specific variables, and scoring method used add noise to the operationalization of overclaiming and the interpretation of the findings. As demonstrated in this investigation and others, overclaiming behavior can be a predictor of negative outcomes. It is important to understand what motivates overclaiming behavior so that researchers can then shift their focus to create intervention and/or prevention programs to reduce overclaiming behavior and, consequently, the negative outcomes it predicts.

### Limitations

Our research certainly comes with limitations. The first and most critical is the two samples collected were of students. Future research should recruit a population with higher levels of religiosity. Another limitation is our one-item measure of religiosity. Previous research has shown that with respect to terrorist activity, religiosity, religious attendance, and prayer all have separate effects (Ginges et al., 2009). Thus, future research should examine these three components separately. Finally, the cultural backgrounds of our samples were mixed, which may have affected some of the results. Study 1 participants were recruited from a region of the United States that borders Mexico. Mexico is predominantly Catholic and characterized by higher cultural masculinity norms when compared to the United States (Hofstede, 2016). These norms may have influenced how religious overclaiming was expressed among men and shaped women’s perceptions of devotion to a partner. Although the diversity between our studies was a strength, it may have led to inconsistent gender interactions. However, it is important to note that our primary hypothesis—that overclaiming would predict partner abuse—was supported across these different samples. Finally, research is needed to identify what characteristics are critical to the core of religious overclaiming and test the construct validity of the B-OCQ. We argue that the B-OCQ is partly capturing a lack of intellectual humility concerning one’s religious knowledge.

### Implications and Future Directions

In addition to offering a potential explanation for the religion-violence paradox, our findings also add to our understanding of how facets of people’s religion can impact their experience of IPV. Additional research can result in the creation of intervention and/or prevention programs such as working with religious authority figures (e.g., priests) to prioritize the safety of victims of IPV. Additionally, our participants across both studies were predominantly Hispanic, which is an understudied population in IPV and a concern in the current state of the field that studies IPV (Bender, 2017). Increasing our cultural competence—our knowledge of cultures other than our own—can help us better serve victims of IPV by tailoring services to assist victims of all backgrounds.

Future studies should focus on identifying why people engage in overclaiming behavior. We mentioned multiple mechanisms and specific variables that could explain overclaiming behavior. In our opinion, intellectual humility is among the most promising lines of research to pursue. Recent research has found that both a lack of intellectual humility and creative curiosity are uniquely linked with overclaiming (Zedelius et al., 2022). Intellectual humility is distinct from Honesty/Humility as defined by the HEXACO assessment both theoretically and operationally (Zedelius et al., 2022). Whereas honesty/humility as defined by HEXACO is focused on interpersonal manipulation and callousness (Book et al., 2015), intellectual humility is focused on the ability to admit you were wrong (Hoyle et al., 2016). Thus, it appears that there are at least two potential paths to overclaiming, which involve curiosity/openness to experience and an inability to admit being incorrect (Zedelius et al., 2022). Future studies should also distinguish between violent behaviors meant to hurt others and violent behaviors in self-defense. It is possible that violence committed in self-defense would have a different relationship with overclaiming. Leach et al, (2008) showed that religion decreases the likelihood of initiating aggression but does not influence the likelihood of responding with aggression if an aggressive act was done to them first. Asking participants to distinguish between violent behaviors perpetrated in self-defense and those not done in self-defense could also help in adequately testing for gender differences in the perpetration of IPV motivated by religious overclaiming. Future studies should also differentiate between intrinsic and extrinsic motivations to be religious. Considering that overclaiming can be a form of self-enhancement, this distinction would be important and would help in more adequately testing for the moderation effects of religiosity in the religious overclaiming–IPV association. Finally, future studies should focus on creating experimental studies to test the causal relationship between religious overclaiming and IPV. These studies were cross-sectional and thus it is hard to establish a causal relationship between overclaiming and perpetration of IPV as well as how gender and religiosity moderate this relationship. The perpetration of IPV may be the result of overclaiming or overclaiming may be the result of knowing that IPV is not socially acceptable.

## Conclusion

The relationship between religion and violence has presented a paradox for researchers. Religion at times is associated with communion and other times with violence toward others. The key to understanding these nuances is perhaps not asking whether religion causes violence, but what facets of religion encourage people to use religion as a justification for violence. This study focused on testing how the tendency to overclaim religious knowledge was associated with the perpetration of IPV in romantic relationships. Our findings indicate that greater overclaiming is associated with greater perpetration of IPV. There were mixed findings for gender, and religiosity was unassociated with the perpetration of IPV.

## Notes

1. Identification with male or female as their gender was important for this study because one of the purposes of this investigation was to test for gender differences among religious overclaimers.

2. The availability of measures in English and Spanish was particularly important for a secondary study that was being run at the same time as Study 1. Therefore, only in Study 1, measures were available in both languages. However, students in the predominantly Hispanic serving institution are bilingual and it is more common for them to complete research studies in English than Spanish.
